# Coaxial helium electrospray for single-particle imaging at X-ray free electron lasers

**DOI:** 10.1107/S1600577525003686

**Published:** 2025-06-06

**Authors:** Safi Rafie-Zinedine, Joachim Schulz, Johan Bielecki, Michael Heymann

**Affiliations:** a European XFEL Holzkoppel 4 22869Schenefeld Germany; b Institute of Biomaterials and Biomolecular Systems University of Stuttgart Pfaffenwaldring 57 70569Stuttgart Germany; Paul Scherrer Institut, Switzerland

**Keywords:** coaxial electrospray, aerosol injection, 3D printing in microfluidics, single-particle imaging, XFELs

## Abstract

Single-particle imaging at X-ray free-electron lasers relies on suitable sample injection of nanoscale macromolecules and particles into the gas phase at room temperature. A coaxial liquid-sheet strategy considerably extended the range of suitable samples to include conductivities from zero to 40000 µS cm^−1^ – a more than about an eightfold increase in range compared with conventional electrosprays. A helium chamber atmosphere in combination with an engineered gas-sheet protected aerosol formation against corona discharge and reduced background noise more than threefold. These results suggest new avenues to qualify ever more demanding biological and material science samples for single-particle imaging in the future.

## Abstract 2

This study presents the Coaxial Helium Electrospray (CHeES) technique, employing a 3D-printed microfluidic coaxial nozzle to broaden the single particle imaging sample range at XFELs, enhancing sample conductivity support and reducing background noise.

## 1. Introduction

The brilliance of X-ray free-electron lasers (XFELs) has paved the way for single-particle imaging (SPI) to capture room-temperature diffraction patterns of nanoscopic particles at high resolution, including biological samples (Sun *et al.*, 2018 ▸; Bielecki *et al.*, 2020 ▸). Their short pulse duration of ∼10 fs is invaluable for resolving ultrafast structural dynamics but also for outrunning conventional radiation damage via ‘diffraction-before-destruction’ imaging (Neutze *et al.*, 2000 ▸). While diffraction patterns generally provide only a 2D image plane of the 3D particle, a complete 3D structure can be reconstructed from combining multiple individual diffraction patterns into a 3D dataset in reciprocal space. Phase retrieval algorithms can then reconstruct the 3D particle structure (Paganin, 2013 ▸). To do so requires individual particles to be as homogeneous and reproducibly delivered as possible, which prompted a continuous evolution in liquid jet and gas phase injection technologies (Knoška *et al.*, 2020 ▸; Bielecki *et al.*, 2019 ▸).

Electrospray has proven especially effective for SPI (Bielecki *et al.*, 2019 ▸). Here, electrical forces counterbalance liquid surface tension to form a cone-jet meniscus called a Taylor cone (Zeleny, 1914 ▸; Chen *et al.*, 1995 ▸; Fu *et al.*, 2011 ▸) that subsequently breaks into a monodisperse stream of nano- to micrometre-sized droplets (Rafie-Zinedine *et al.*, 2024 ▸). While traveling to the X-ray interaction region, solvent evaporates to yield virtually contaminant-free particles in the gas phase (Bielecki *et al.*, 2019 ▸), which affords high scattering contrast and minimal diffraction background signals. Particles are neutralized to limit their capture on grounded surfaces and focused to a particle beam into the X-ray interaction region via an aerosol lens stack (De La Mora & Riesco-Chueca, 1988 ▸; Liu *et al.*, 1995 ▸; Hantke *et al.*, 2018 ▸). Aerodynamic lensing can accelerate particles to hundreds of metres per second (Hantke *et al.*, 2018 ▸; Bielecki *et al.*, 2019 ▸). This delivery modality is hence particularly well suited to prevent multiple exposures of the same particle at fast megahertz pulse rates, such as those achieved at the European XFEL, which emits X-ray trains at 10 Hz, each comprising up to 2700 pulses, spaced 220 ns apart (Decking *et al.*, 2020 ▸). To date, a broad range of samples, including cells (Schot *et al.*, 2015 ▸), cell organelles (Hantke *et al.*, 2014 ▸), viruses (Seibert *et al.*, 2011 ▸; Ekeberg *et al.*, 2015 ▸; Kurta *et al.*, 2017 ▸; Rose *et al.*, 2018 ▸; Munke *et al.*, 2016 ▸; Reddy *et al.*, 2017 ▸; Daurer *et al.*, 2017 ▸; Sobolev *et al.*, 2020 ▸; Gorkhover *et al.*, 2018 ▸; Lundholm *et al.*, 2018 ▸), proteins (Ekeberg *et al.*, 2024 ▸) and inorganic nanoparticles (Ho *et al.*, 2020 ▸; Ayyer *et al.*, 2021 ▸) have already been imaged using this approach.

While providing encouraging proof-of-principle results, many biological samples continue to be difficult to electrospray for SPI. Firstly, the optimal salt concentration for the sample may be too far away from the one required for a stable Taylor cone, forcing non-ideal trade-offs. In particular, low salt regimes fail to sufficiently suppress surface tension forces for reliable injection. A co-flow regime, where the low conductivity sample is surrounded by a suitable electrospray buffer, could provide the optimal conditions for both sample stability and Taylor cone formation. A similar double-flow focusing strategy has already been demonstrated for gas dynamic virtual nozzles to inject microcrystal slurry (Oberthuer *et al.*, 2017 ▸; Knoška *et al.*, 2020 ▸). Also, non-conductive olive oil, which alone cannot electrospray (Chen *et al.*, 1995 ▸), could be aerosolized by co-injection with a conductive water solution in a coaxial electrospray (Loscertales *et al.*, 2002 ▸). Here, a Taylor cone formed by the water solution was coated with a thin layer of olive oil to produce a core-shell capsule aerosol. Secondly, background scattering from heavier gases such as N_2_ and CO_2_ used in the electrospray chamber result in too low signal-to-noise ratios for structure determination (Ekeberg *et al.*, 2024 ▸), especially for smaller particles like single proteins or viruses below 50 nm diameter. A recently proposed helium electrospray (HeES) method introduced helium as the main gas for particle transport, while restricting N_2_ and CO_2_ to a narrow region around the Taylor cone to protect it from corona discharge, thereby decreasing background noise significantly (Yenupuri *et al.*, 2024 ▸).

While conceptually simple, realizing such filigree coaxial sheet flows for both the liquid and the gas stream experimentally requires precisely microfabricated injectors. Cutting-edge two-photon polymerization has been shown to be capable of realizing complex 3D fluidic devices with sub­micrometre precision. This has been readily exploited in engineering microfluidic nozzles, offering improved performance, miniaturization and customization (Knoška *et al.*, 2020 ▸; Vakili *et al.*, 2022 ▸).

**Figure 1:**
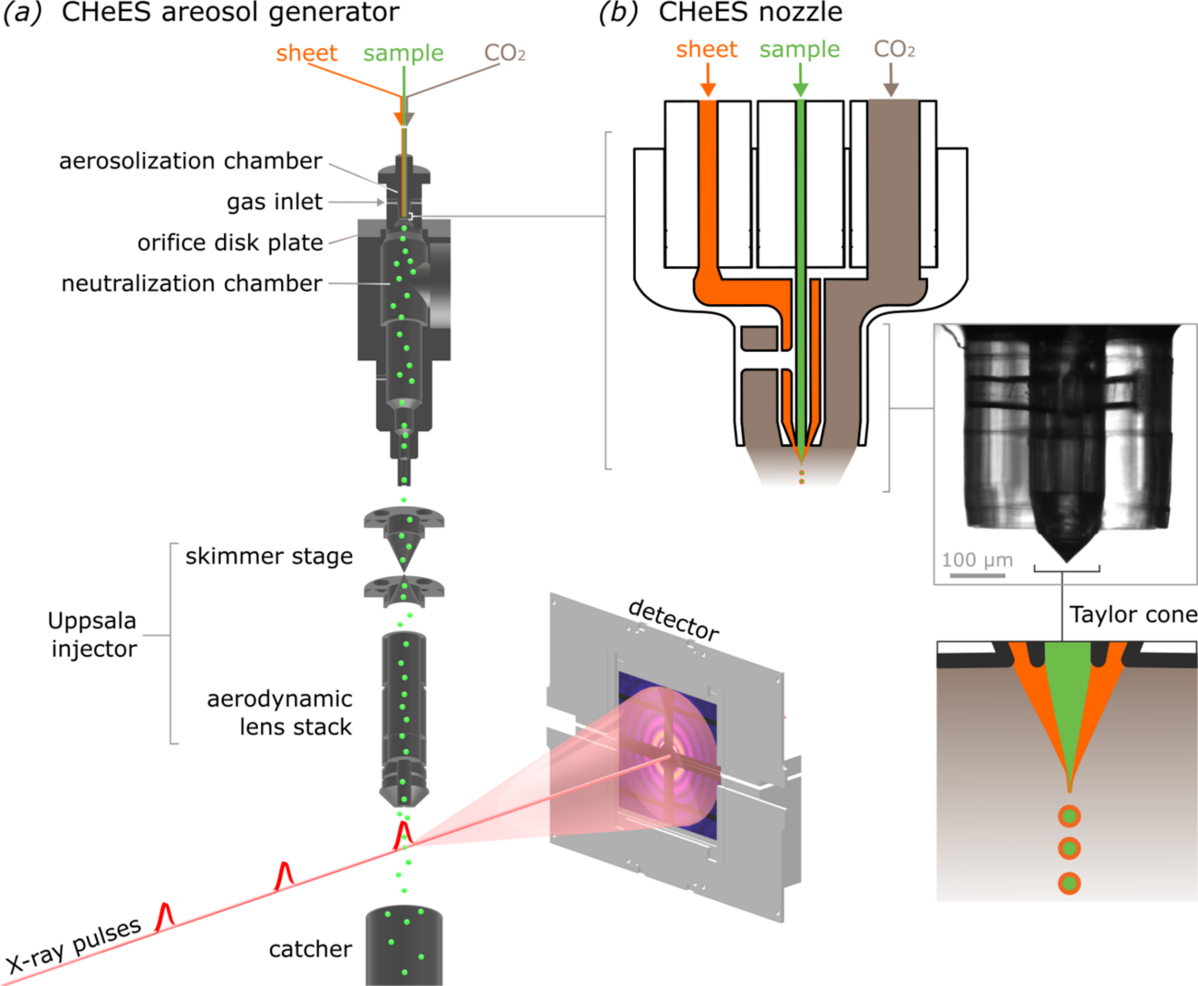
Schematic diagram of the coaxial helium electrospray (CHeES) aerosol injector. (*a*) After aerosolization in the CHeES nozzle, the resulting particle beam is neutralized, passes a skimmer stage and is focused by an aerodynamic lens stack into the X-ray interaction chamber to collect diffraction patterns. (*b*) The CHeES nozzle is designed to initiate a coaxial sample and electrospray buffer flow in the Taylor cone under a CO_2_ gas sheet atmosphere to protect aerosol formation against corona discharge.

We present a coaxial helium electrospray (CHeES) nozzle for SPI at XFELs. It handles a broader range of sample conductivities, with lower background noise than traditional electrospray injectors (Fig. 1 ▸). CHeES features a compact 3D nozzle that delivers two coaxial liquids and a coaxial gas to ensure stable sample delivery. It accommodates samples from non-conductive to high conductivities up to 40000 µS cm^−1^, while N_2_ and CO_2_ gas loads are reduced by 3.5 and 2.2 times, respectively. CHeES readily integrates with existing experimental setups, including the ‘Uppsala’ injector at the SPB/SFX instrument of the European XFEL. CHeES realized a more than threefold reduction in background noise without affecting injection yield, demonstrated experimentally by high-quality diffraction patterns from 55 nm cube-shaped silver nanoparticles. CHeES could hence enable the study of structures previously thought too difficult or impossible to resolve in SPI.

## 2. Methods

### 2.1. Simulations of gas flow around the Taylor cone

To optimize the gas distribution around the Taylor cone, the fractional concentration of each gas *x*_*i*_ was determined through finite element modeling (SI-Figure 1) using *COMSOL Multiphysic*s (Version 6.2), following the method described by Yenupuri *et al.* (2024 ▸). Briefly, gas flow dynamics within the aerosolization chamber were modeled using a laminar flow interface, which computed velocity and pressure. Simultaneously, a transport interface was employed to evaluate the behavior of gaseous mixtures by calculating their mass fractions. The gas mass fractional concentration was defined as
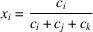
where *c*_*i*_ represents the molar concentration of the specific gas with fractional concentration *x*_*i*_, and *c*_*j*_ and *c*_*k*_ are the molar concentrations of the other two gases present in the mixture.

### 2.2. CHeES design

**Figure 2:**
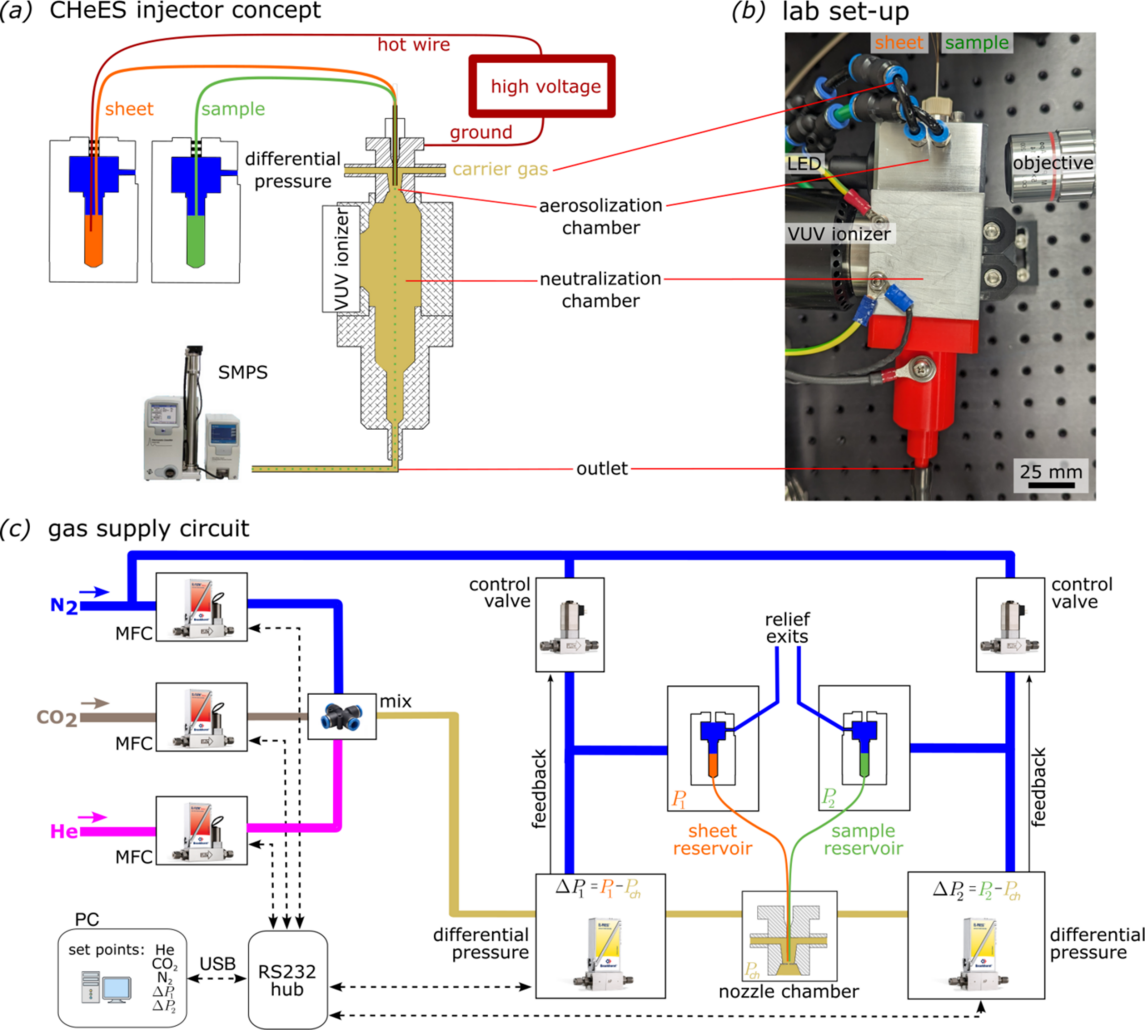
CHeES aerosol generator setup. (*a*) A detailed illustration of the CHeES system configuration shows the liquid lines, high voltage and carrier gas connections. The coaxial sheet (outer liquid) and sample (inner liquid) are aerosolized through Taylor cone formation and travel to the neutralization chamber, where they are neutralized by a vacuum ultraviolet neutralizer before exiting into an SMPS for droplet sizing. (*b*) Photograph of the physical configuration of the CHeES aerosolizer in the laboratory. (*c*) Schematic of the gas flow circuit with an emphasis on the integration of mass flow controllers and differential pressure meters coupled with control valves to regulate the flow rates of CHeES liquids and carrier gases.

The CHeES nozzle was designed in Siemens’ *NX* CAD-software and exported to STL file format. The design is available as supporting information (SI-Figure 2) and from the GitHub repository (https://github.com/safirafie/ESDesign). Nozzles were 3D printed as previously reported (Knoška *et al.*, 2020 ▸; Vakili *et al.*, 2022 ▸). In brief, IP-S photoresist was exposed on a Photonic Professional GT equipped with a 25× objective using solid-volume settings with 1 µm slicing and 0.5 µm hatching when configuring the print job in DeScribe (all Nanoscribe GmbH). Excess resin was developed away by incubating in propylene glycol methyl ether acetate (PGMEA) for about one day, followed by three 30 minutes wash steps in 2-propanol and subsequent air-drying on a cleanroom cloth. Nozzles were then placed on a flat polydimethylsiloxane (PDMS) sheet to insert three fused silica capillaries (Polymicro tubing, Molex Inc.), each with a 360 µm outer diameter (OD) and inner diameters (IDs) of 40 µm for the sample, a 75 µm for the buffer, and 220 µm for CO_2_ gas with a length of approximately 40 cm. Devcon 5-minute epoxy was used to glue all inlet capillaries into the nozzle body and subsequently into a hollow stainless-steel tubing (U-145, ID 0.046 inches, OD 1/16 inches, IDEX).

### 2.3. Experimental setup

Our SPI setup at European XFEL is based on a previous instrument (Rafie-Zinedine *et al.*, 2024 ▸) with a modified electrospray aerosol generator (Fig. 1 ▸). In brief, the CHeES nozzle aerosolizes a sample solution into positively charged droplets. Solvent evaporation reduces these into positively charged particles, which enter a 25.2 mm-diameter neutralization chamber with a vacuum ultraviolet ionizer (Hamamatsu L12542). The ionizer radiation creates bipolar gas ions that neutralize the charged particles to prevent their adhesion to the grounded chamber surfaces. Neutralized particles then pass through the ‘Uppsala’ injector (Hantke *et al.*, 2018 ▸), which includes a two-skimmer box for excess gas removal, followed by an aerosol lens stack for particle focusing. These particles intersect with a pulsed X-ray beam and the resulting diffraction patterns are captured by a downstream detector. In addition, a residual gas analyzer can monitor the gas composition within the interaction chamber in real time (Mancuso *et al.*, 2019 ▸).

A three-way gas flow control circuit is used to form a defined CO_2_ gas sheet around the Taylor cone (Fig. 2 ▸). This configuration can utilize a nitrogen or helium mode to prevent corona discharge (Table 1 ▸). Mass flow controllers (F-201CV, Bronkhorst) regulate each gas flow rate independently. Differential pressure meters (P-506C, Bronkhorst) with control valves (F-001, Bronkhorst) maintain a constant pressure difference Δ*P* across the capillaries to regulate sample and sheet-liquid flow rates independently. A microscope comprising a fiber-coupled LED, infinity-corrected objectives (5× Plan Apo, *f* = 200, Mitutoyo), a tube lens (TTL200-A, Thorlabs) and a CCD camera (acA2440-2gm, Basler) was used to image the jetting dynamics at the nozzle orifice. To induce Taylor cone formation, high voltage was applied to the sheet-liquid reservoir, while the orifice plate, aerosolization and neutralization chambers were grounded. The CHeES nozzle tip and the grounded orifice plate were placed approximately 1 mm apart. Droplet sizes were characterized using a scanning mobility particle sizer (SMPS) (3082 electrostatic classifier and 3788N-WCPC, TSI), linked to the generator outlet to record size distributions.

Each experimental data point sampled coaxial-liquid-sheet and sample flow rates repeatedly, including technical replicates with different 3D-printed CHeES nozzles of the same design. Real-time microscopy monitored Taylor cone stability to ensure reproducible data collection. Gas mass flows were limited to not exceed 2 × 10^−4^ mbar chamber pressures to protect the AGIPD X-ray detector (Mancuso *et al.*, 2019 ▸). Other applications, not limited in this regard, are expected to achieve higher transmission efficiencies for increased gas flows (Fu *et al.*, 2011 ▸).

Core-shell droplet size quantification experiments were performed using a reference sample solution containing 1% (*v*/*v*) sucrose with 20 m*M* ammonium acetate in water. Conductivity samples ranged from 0 m*M* (0 µS cm^−1^) to 550 m*M* ammonium acetate (∼44000 µS cm^−1^). Buffer conductivities were measured with a conductivity meter (SevenExcellence Cond meter S470, Mettler Tolledo). Droplet sizes were derived from SMPS-measured particle sizes as previously reported (Bielecki *et al.*, 2019 ▸). For this, sucrose was used as a non-volatile additive to determine the droplet size as
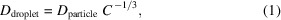
with *D* the respective diameters and *C* the concentration (vol/vol) of sucrose.

### 2.4. CHeES operating conditions

Sample flows ranged between 32 and 877 nL min^−1^ and coaxial-sheet flows between 401 and 602 nL min^−1^. Flow rates (*Q*) were regulated by adjusting the pressure difference (Δ*P*) across the capillaries, in accordance with the Hagen-Poiseuille equation,
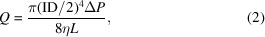
with η the fluid viscosity, inlet dimensions of *L* = 40 cm and inner diameters of 40 µm for sample and 75 µm for the coaxial-sheet. A voltage in the range 2–4 kV relative to the counter electrode was applied to the coaxial-sheet solution to induce and stabilize the Taylor cone. Formation and stability of the Taylor cone were observed visually via the in-line microscope and by tracking the applied electric current to adjust the voltage as needed. Note that a stable Taylor cone constitutes an equilibrium between electrostatic forces and liquid surface tension at the interface (Zeleny, 1914 ▸; Taylor, 1964 ▸). Also, the CHeES configuration does not require an electrode to apply a voltage on the sample inlet. Under these conditions, the Taylor cone is formed by the outer coaxial-sheet liquid, which envelops the inner sample liquid stream. This results in the ejection of positively charged composite droplets, consisting of a sheet-buffer and a sample liquid, as illustrated in Fig. 1 ▸. After droplet ejection, all volatile components from both liquid streams evaporate, leaving behind sample particles in the gas phase.

For stable injection, the helium mode light gas mixture was slightly adjusted for water or ethanol liquid-sheets (Table 1 ▸). Helium was preferred over the highly flammable hydrogen. Alternatively, the injector can also be operated with the conventional carrier gas environment (nitrogen mode). While a pure nitrogen atmosphere could not prevent corona discharge, inclusion of minimal quantities of CO_2_ sufficed to maintain stable electrosprays.

## 3. Results and discussion

### 3.1. Multiphysics modeling of gas sheet for corona discharge protection

The high voltages required for Taylor cone formation pose a significant risk of corona discharge. Once an electric field exceeds the breakdown voltage, the immersion gas atmosphere itself becomes conductive, as gas molecules ionize. Arcing within the experimental chamber can irreparably damage sensitive electronic components, such as the detector. Also, a loss of the positively charged liquid surface inadvertently destabilizes the Taylor cone.

While light gases such as helium or hydrogen show lower X-ray background scattering than heavier gases such as nitrogen and carbon dioxide, their lower electric field thresholds (Paschen, 1889 ▸) have so far been considered too risky for electrospray-based SPI.

**Figure 3:**
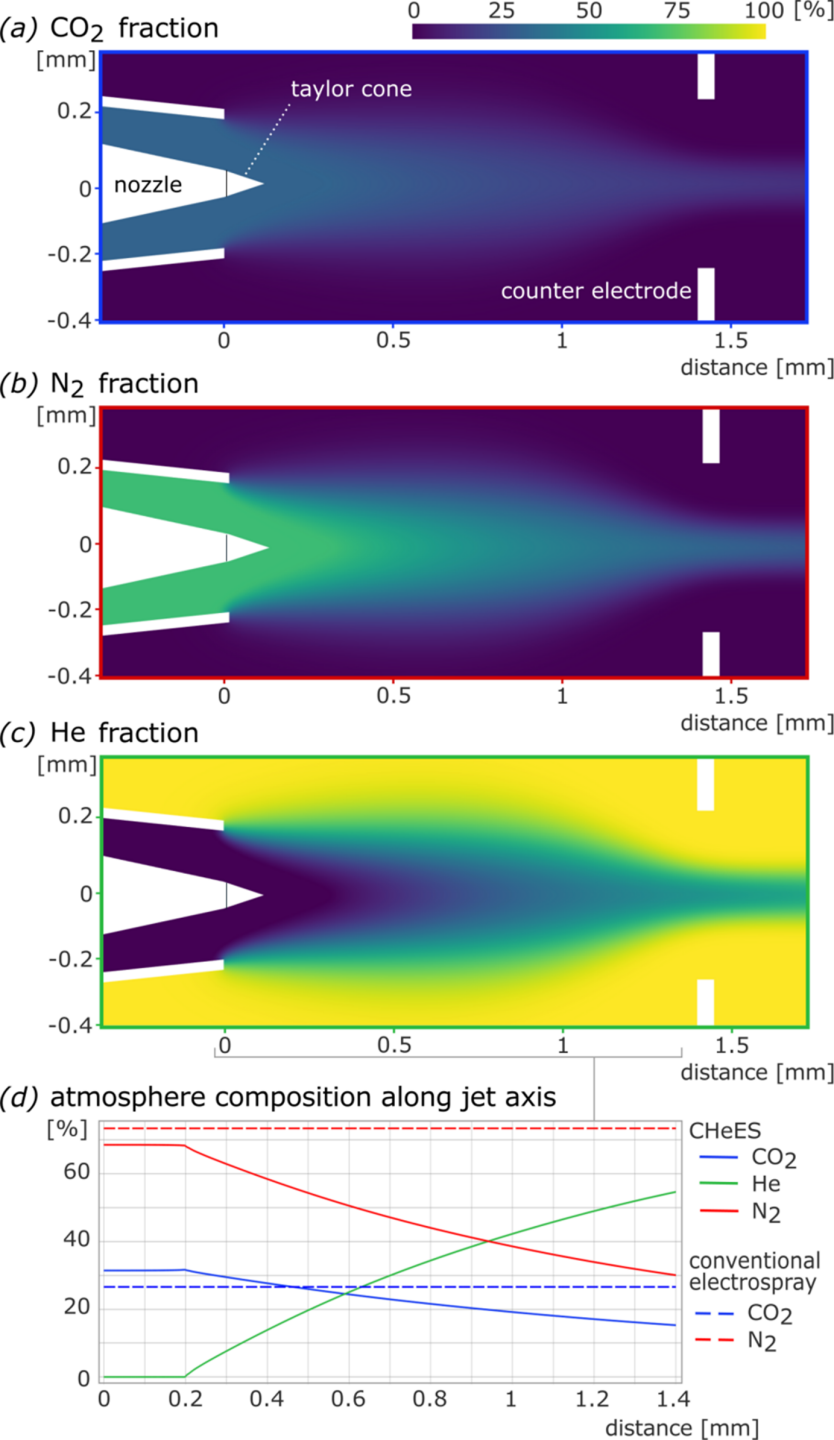
Finite element modeling of gas flows around the Taylor cone in the CHeES nozzle underscore the effectiveness of gas shielding to prevent corona discharge. Fractional gas concentrations of (*a*) CO_2_, (*b*) N_2_ and (*c*) helium indicate that a large protective CO_2_ sheet can form around the Taylor cone. (*d*) A safe gas atmosphere composition similar to the conventional electrospray injection extends about half way from the tip of the Taylor cone to the counter electrode.

**Figure 4:**
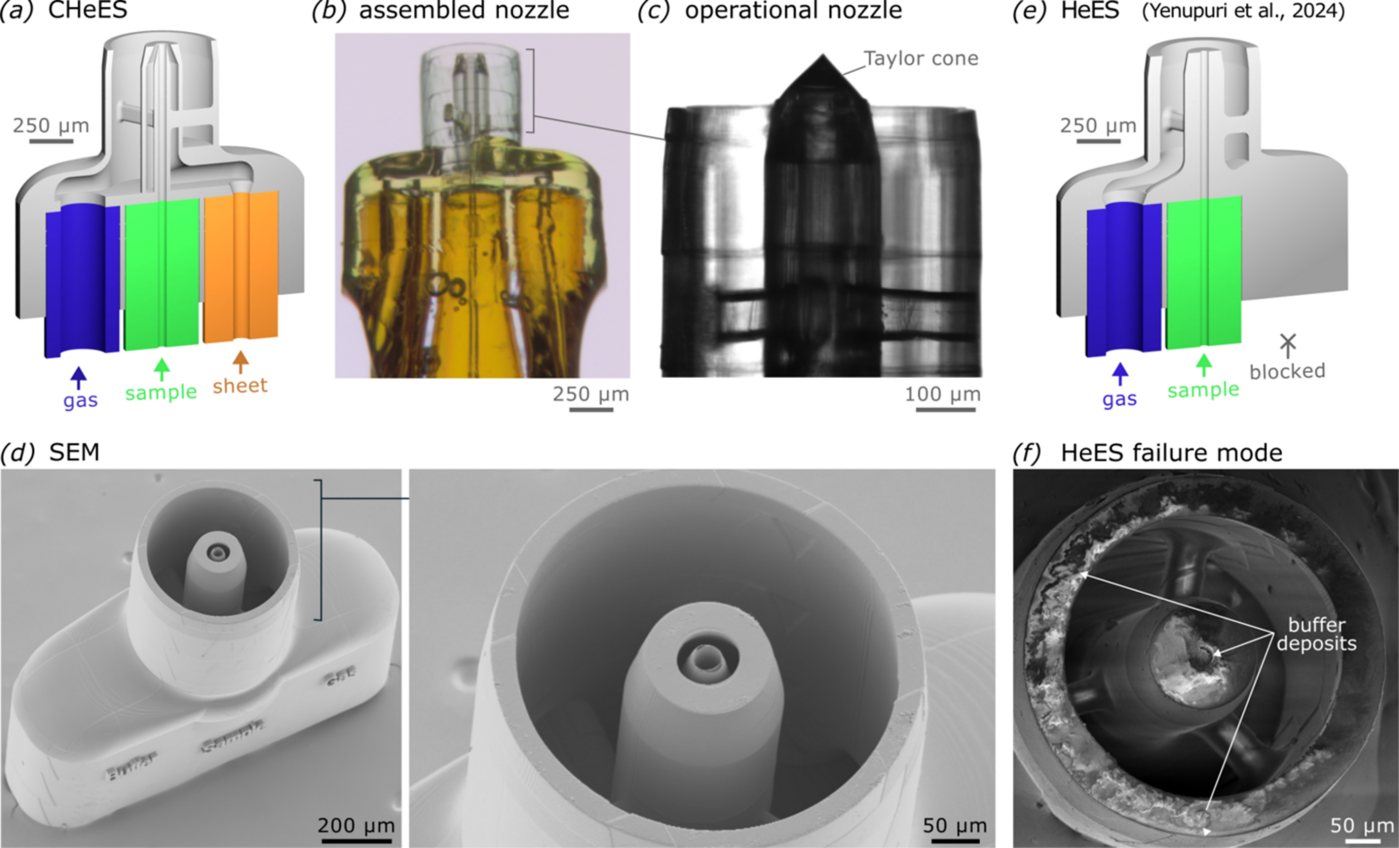
Design and a ssembly of the CHeES nozzle. Narrow 40 µm and 75 µm inner diameter lines minimize inner sample and outer coaxial-liquid-sheet dead volumes. (*a*) CAD-design with color coded inlet capillaries and (*b*) stereomicrograph of an assembled nozzle. (*c*) Bright-field microscopy image of a CHeES nozzle in operation, forming a Taylor cone at the nozzle orifice. (*d*) Scanning electron microscopy confirmed accurate fabrication of the fine features of the nozzle orifice used to form a conductive liquid sheet around the sample stream in the Taylor cone, giving CHeES more stable operating characteristics compared with conventional electrospray nozzles (*e*), which are prone to clogging from non-volatile solutes deposition on the orifice over time. Scanning electron micrographs indicate extensive sucrose deposits (*f*) on the orifice after clogging from spraying 1% *v*/*v* sucrose buffer for about an hour.

We considered the conventional nitrogen chamber atmosphere comprising 73% N_2_ and 27% CO_2_ around the Taylor cone as a safe reference (Table 1 ▸). Finite element modeling confirmed that a comparable gas atmosphere can be maintained to about half a millimetre away from the Taylor cone by introducing a narrow gas-sheet of 30 ml min^−1^ CO_2_ directly around the Taylor cone at the CHeES orifice to protect against corona discharge (Fig. 3 ▸, SI-Figure 3). With steady-state injection of 3 L min^−1^ helium and 60 ml min^−1^ nitrogen, this would induce a 65% helium atmosphere into the X-ray interaction region for minimal background scattering. High pressures remain effectively confined to the nozzle orifice region and quickly dissipate after the grounded plate orrifice (SI-Figure 4).

### 3.2. CHeES nozzle design and characterization

Considering these simulation results, we designed a nozzle of about 1.4 × 0.6 × 1.25 mm in size (Fig. 4 ▸, SI-Figure 2). The design features three capillary inlets for 360 µm OD fused silica capillaries that propagate into the previously simulated coaxial hydrodynamic flow focusing geometry. It was readily microfabricated using established two-photon stereolithography and assembly techniques (Knoška *et al.*, 2020 ▸; Vakili *et al.*, 2022 ▸) and achieved a stable cone-jet while spraying buffers of 80 m*M* ammonium acetate in ethanol or 20 m*M* ammonium acetate in water (Fig. 4 ▸*d*). The coaxial liquid sheet completely eradicated nozzle clogging artifacts from solute crust formation at the liquid–gas interface, which represents a common failure mode in conventional electrosprays such as the related HeES nozzle (Yenupuri *et al.*, 2024 ▸) (Figs. 4 ▸*e* and 4*f*). The operating conditions in the helium and nitrogen mode (Table 1 ▸) were slightly adjusted for HeES (SI-Table 1).

### 3.3. Coaxial liquid sheet formation and conductivity range quantification

**Figure 5:**
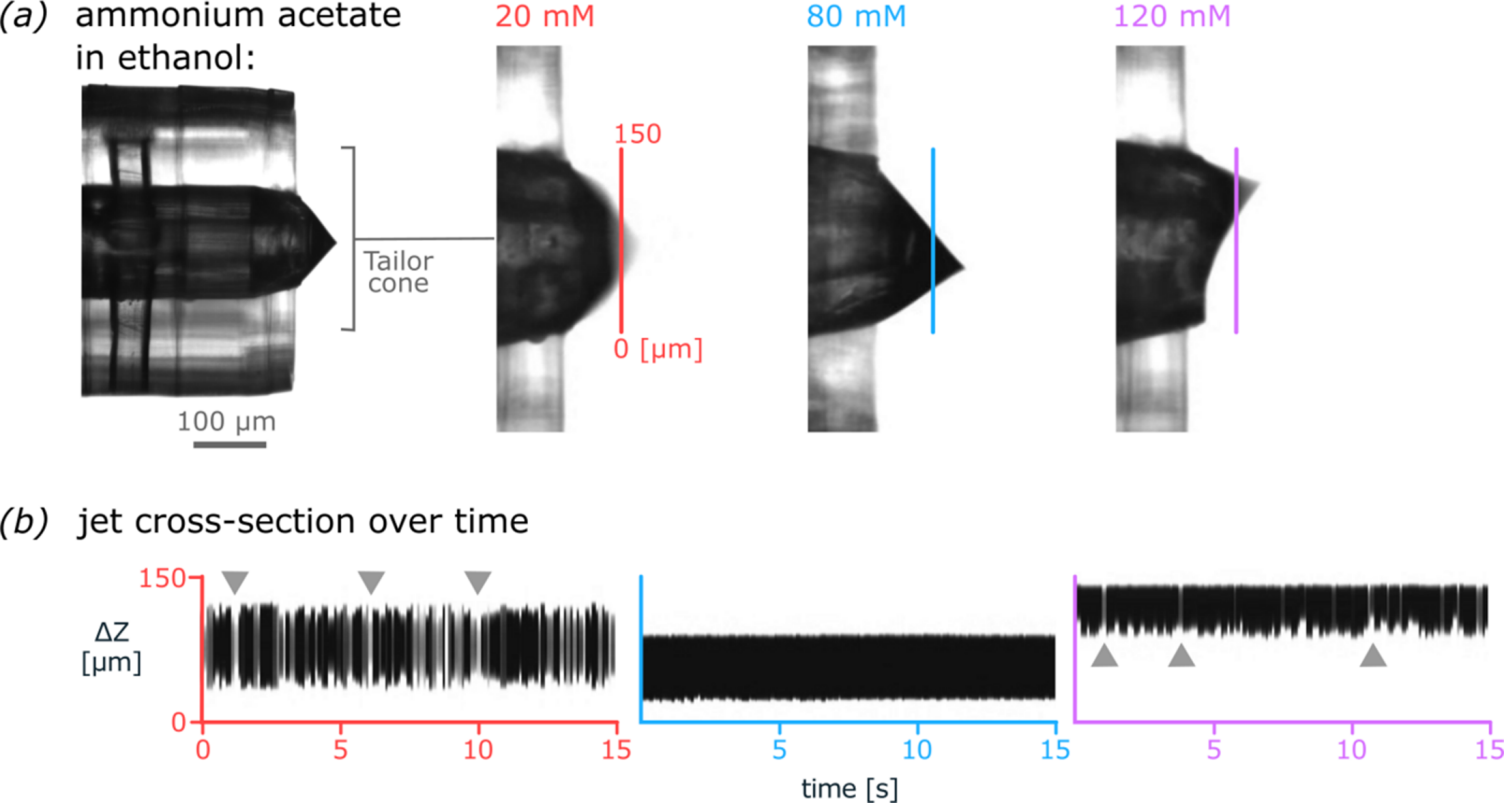
Coaxial-sheet-liquid optimization for CHeES Taylor cone stability. (*a*) Among the tested range of 20–120 m*M* ammonium acetate, an 80 m*M* ammonium acetate in ethanol showed optimal Taylor cone stability and aerosol generation when injected at 401 nL min^−1^ (SI-Movie 1). (*b*) A representative cross-section of the Taylor cone was recorded over a duration of 15 s. Gray arrows indicate strong cone shape fluctuations for 20 m*M* and 120 m*M* ammonium acetate in ethanol.

An ideal liquid-sheet combines a low surface tension with a suitably large conductivity to form a stable Taylor cone at low electric fields. This in turn allows for lower applied voltages and hence overall lower gas-sheet flows around the Taylor cone, as the critical breakdown voltage described by Paschen decreases (Paschen, 1889 ▸). Following the original double flow-focusing concept (Oberthuer *et al.*, 2017 ▸), we explored ammonium acetate in ethanol as a low surface tension coxial-sheet buffer by testing against a 1% *v*/*v* sucrose solution in water as the inner liquid. We identified a 401 nL min^−1^ sheet of 80 m*M* ammonium acetate in ethanol with a conductivity of 659 µS cm^−1^ to show best Taylor cone stability and aerosol generation (Fig. 5 ▸, SI-Movie 1). Helium mode (Table 1 ▸) was defined to stably jet with this ideal sheet liquid. For comparison, a 5 m*M* ammonium acetate in water with a comparable conductivity of 503 µS cm^−1^ liquid-sheet flown at 357 nL min^−1^ required a much higher 4200 V for stable jetting. Accordingly, heavy gas flows were increased to 80 ml min^−1^ of N_2_ and 60 ml min^−1^ of CO_2_ gas-sheet in helium mode for water-based coaxial-liquid-sheets (Table 1 ▸).

**Figure 6:**
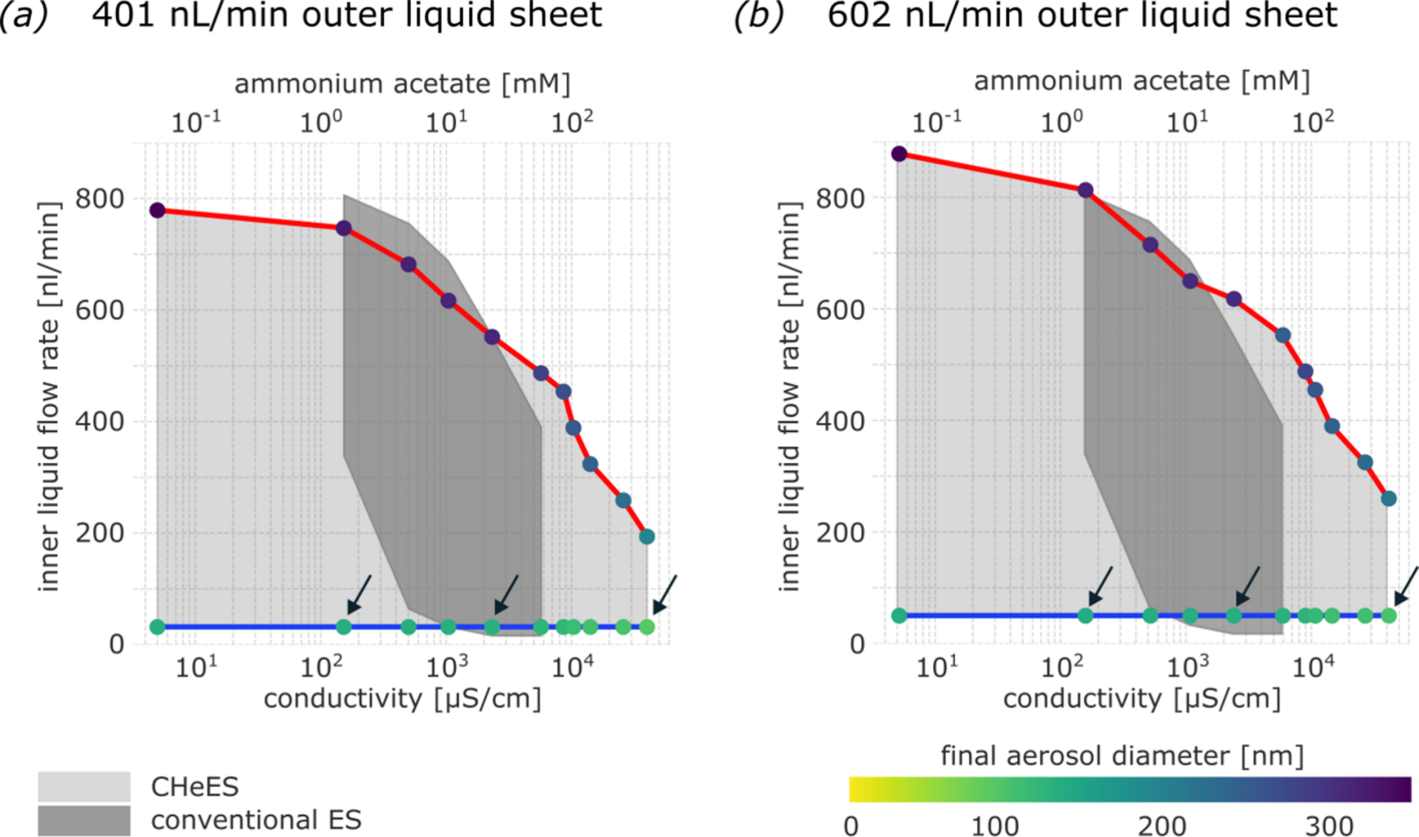
Conductivity range for effective aerosol generation by the CHeES injector. A wide spectrum of sample conductivities from 0 to 40000 µS cm^−1^ could be injected, exceeding the conventional electrospray system. Adjusting the outer liquid flow rates from (*a*) 401 nL min^−1^ to (*b*) 602 nL min^−1^ affected minimal and maximal flow rates for the inner fluid only marginally. Inner droplet sizes of 60–340 nm diameter were aerosolized in both cases, with an inverse correlation against sample conductivity. Three conditions covering low-, mid- and high-conductivity regimes, here indicated by black arrows, were selected for a detailed volume analysis for both outer flow rates tested.

To test our hypothesis for liquid sheet assisted Taylor cone formation, we titrated inner sample conductivity against an outer liquid-sheet reference of 80 m*M* ammonium acetate in ethanol. We then quantified the minimum and maximum inner flow rate limits for stable Taylor cone formation at fixed outer flow rates of 401 or 602 nL min^−1^ (Fig. 6 ▸). Inner sample liquid containing 1% sucrose in deionized water, while non-conductive with 0 µS cm^−1^, achieved a maximum stable flow rate of ∼800 nL min^−1^. Increased inner sample liquid conductivity resulted in noticeably decreased maximum flow rates, and at 550 m*M* ammonium acetate with ∼44000 µS cm^−1^ the Taylor cone could no longer be stabilized. The CHeES nozzle thus sprayed samples ranging from non-conductive to comparably high conductivities of up to 40000 µS cm^−1^ reliably. In contrast, the conventional electrospray nozzle operated stably only for the much narrower range between 100 and 6000 µS cm^−1^, as unsuitably low or excessively high surface charge densities impede cone-jet stability. We can hence deduce that the coaxial-sheet hypothesis holds to the extent that the range of acceptable sample buffers could be noticeably improved.

Droplet size quantification from SMPS measurements and equation (1) implicated inner liquid flows to contribute droplet volumes ranging from 60 to 340 nm in diameter (SI-Figure 5), which are well suited for SPI. Notably, identical liquid flows for more conductive samples yielded overall smaller droplets. This is consistent with the required balance between liquid surface tension and electric forces for Taylor cone stability, which necessitates a sufficiently large surface charge density that itself is compounded by the liquid’s conductivity.

Overall, the minimum inner liquid flow rate remained insensitive to a wide range of outer sheet liquid conductivities while being more sensitive to the outer liquid flow rates. We expect this to be due to a minimal inner liquid flow rate threshold, below which the outer liquid’s pressure induces the inner liquid to flow backwards into the reservoir.

### 3.4. Analysis of effective volume contribution of inner and outer liquid flows

**Figure 7:**
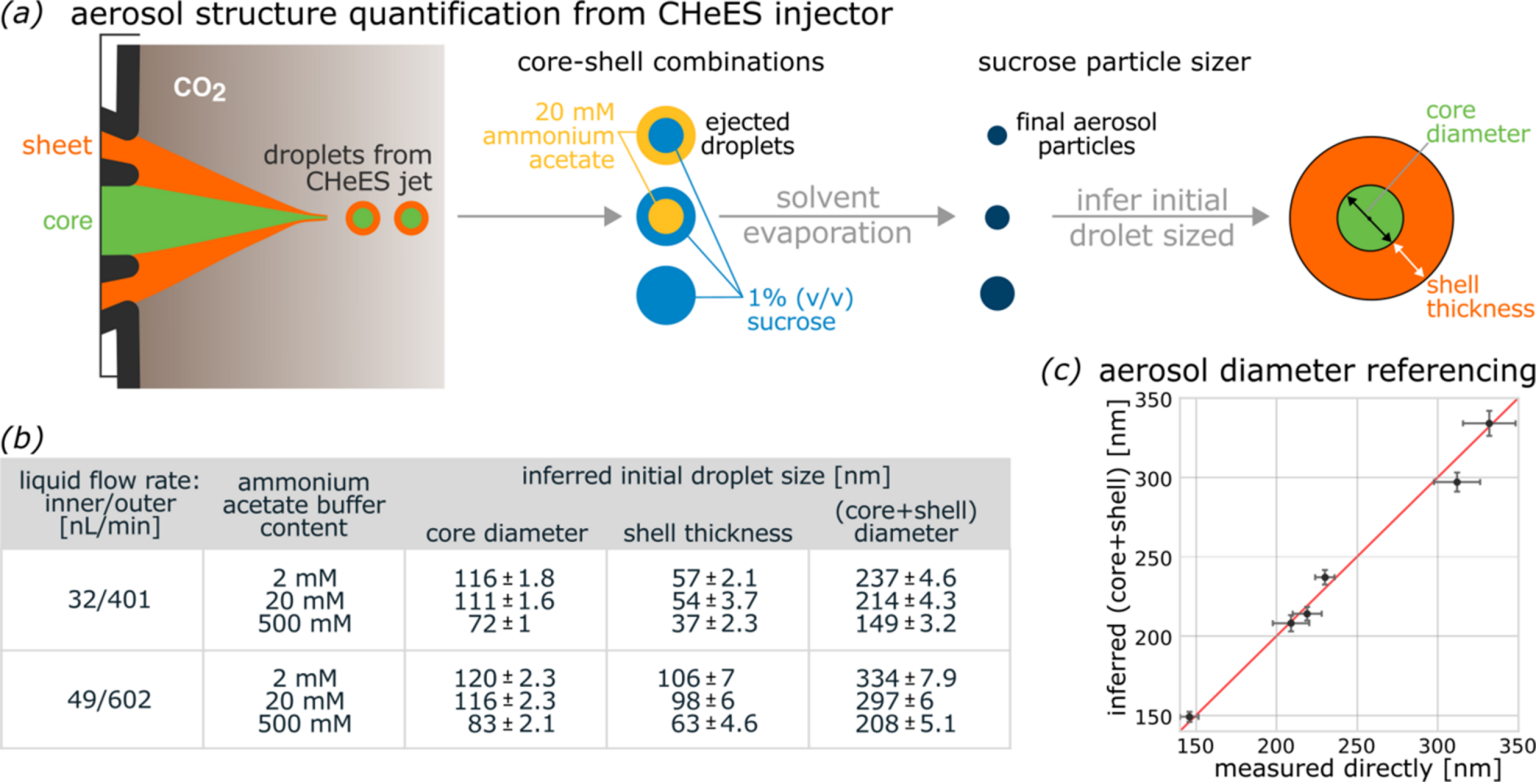
Detailed quantification of droplet structure produced by CHeES. (*a*) Three independent experiments were used to quantify a condition. Either the inner core (top), outer sheet (middle) or both (bottom) portions of the jet are supplemented with sucrose for SMPS particle sizing after solvent evaporation. (*b*) For the six conditions tested, higher surface charge at the Taylor cone produces smaller droplets and thus smaller particles (SI-Table 2). (*c*) Core-shell diameter quantified from separate experiments (inferred) or directly quantified showed very good agreement with each other.

A combinatorial assay was used to quantify the effective volumes contributed to emitted droplets from the inner sample and the outer coaxial-sheet liquid flows in the CHeES injector (Fig. 7 ▸). For this, we focused on six conditions on the lower stable jetting limit – three each for the 401 and 602 nL min^−1^ outer liquid flow rate regimes previously tested (Fig. 6 ▸). For each combination of conductivity and flow rate, we performed SMPS sucrose particle sizing after complete solvent evaporation. A 1% *v*/*v* sucrose solution was introduced into the inner liquid only (1), the outer liquid only (2) or into both (3). From this the core diameter (1), the shell thickness (2) or the complete core-shell droplet (3) could be accessed (SI-Table 2). The smallest droplet diameter produced by CHeES was approximately 149 nm, with a core diameter of about 72 nm and a shell thickness of roughly 37 nm. Size dispersion for each experiment remained below 5%. Furthermore, we noticed a good agreement between directly measured (3) and inferred (sum of 1 and 2) overall diameters (Fig. 7 ▸*c*), confirming the stability and repeatability of the experiments.

### 3.5. Background noise reduction

Based on the previous CHeES injector characterization, we set out to quantify the achievable background noise reduction from a combined coaxial liquid-sheet plus gas-sheet configuration at the SPB/SFX instrument of the European XFEL. For simplicity, we prioritized relating the detector background noise between helium mode in a CHeES nozzle and nitrogen mode used with conventional electrospray nozzles (Table 1 ▸). The optimized coaxial liquid-sheet solution of 80 m*M* ammonium acetate in ethanol was used to help deliver 55 nm silver nanocubes suspended in ethanol at a concentration of 1.16 × 10^11^ particles ml^−1^. Due to a lack of charge carriers, this inner sample would fail to inject in a conventional electrospray (Rafie-Zinedine *et al.*, 2024 ▸), including our HeES injector (Yenupuri *et al.*, 2024 ▸), which features a similar sheet-gas but lacks a coaxial sheet-liquid (Fig. 4 ▸*e*). The injected sample for conventional electrospray hence combined ammonium acetate and silver cube concentrations distributed into both CHeES liquid lines into a single ethanol solution.

**Figure 8:**
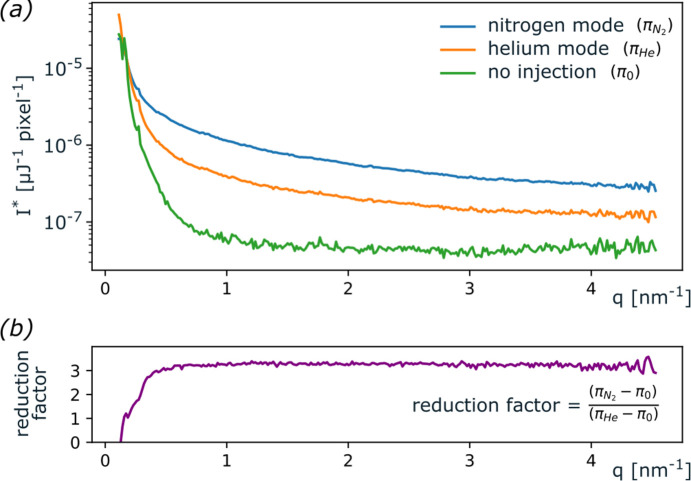
Comparative analysis of radial diffraction background profiles at the European XFEL SPB/SFX instrument. Throughout the varying scattering vectors (*q*), photon counts per µJ per pixel for helium mode (π_He_) were lower than for nitrogen mode (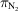), but still noticeably higher than at steady state chamber without injection (π_0_). The background noise reduction for helium mode was quantified as *I*_rel_ = 3.244 ± 0.075 lower then nitrogen mode.

The residual gas analyzer in the SPB/SFX chamber showed reductions in the concentrations of heavier gases: a 3.5-fold decrease for N_2_ and a 2.2-fold decrease for CO_2_ under helium mode with the respective gas-sheet flow. Furthermore, radial profiles of background scattering patterns measured a background noise reduction factor *I*_rel_ = 3.244 ± 0.075 (Fig. 8 ▸). This decrease is consistent with the reduced gas scattering cross-section expected in helium (new) over nitrogen (old) mode. Elastic scattering from a gas molecule is proportional to the square of its number of electrons, *Z*. The chamber atmosphere can then be considered to be the weighted sum over all gas species in the mixture and the background noise reduction factor can be estimated as
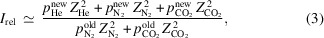
with SPB/SFX chamber partial gas pressures, *p*_gas_, of 3.2 × 10^−6^, 2.7 × 10^−6^ and 1 × 10^−6^ mbar for He, N_2_ and CO_2_ in helium mode, and 9.5 × 10^−6^ and 2.3 × 10^−6^ mbar for N_2_ and CO_2_ in conventional electrospray. We approximate *I*_rel_ ≃ 0.3, corroborating our earlier diffraction pattern based background reduction measurement.

Both configurations achieved a hit rate of approximately 4%, indicating that the CHeES system can match the conventional electrospray system in particle transmission efficiency to the interaction chamber. For reference, SI-Figure 6 presents examples of four high-quality diffraction patterns from this silver nanocube sample using the CHeES system. However, an accurate experimental comparison in this regard is inherently difficult, as the smallest alignment and focus drifts of the nanometre X-ray beam or minute gas flow obstructions from sample depositions along the injection system can quickly deteriorate the signal. A detailed analysis of collected silver cube diffraction signal quality will be reported elsewhere.

## 4. Conclusion

The combination of a coaxial liquid-sheet and gas-sheet into a precision 3D printed nozzle, called CHeES, offers several advantages for SPI. CHeES can emit droplets as small as ∼149 nm in diameter, consisting of a ∼72 nm inner sample liquid compartment enclosed by a ∼37 nm sheet. These are well suited for SPI and comparable with the smallest droplet diameter generated by conventional electrospray (Bielecki *et al.*, 2019 ▸). The previously reported helium gas-sheet injection concept (Yenupuri *et al.*, 2024 ▸) was validated in diffraction experiments to confirm a background reduction and hence a signal-to-noise improvement by a factor of more than threefold. Further optimizing the gas sheet in the future may help lower parasitic gas loads in the experimental chamber. For example, a Laval nozzle taper can help limit the radial dissipation of the gas sheet to further reduce the injection of heavy gas (Gerhardter *et al.*, 2022 ▸).

Even more fascinating is the prospect of decoupling the liquid sample buffer composition from the electrospray ionization process, afforded by the coaxial liquid-sheet. Firstly, the improved long-term jet stability can help pave the way to adopt high-density electrospray nozzle arrays (Kim & Velásquez-García, 2025 ▸) for SPI to increase overall aerosol densities and hence hit-rates in the X-ray interaction region. Secondly, samples with conductivity ranging from zero up to 40000 µS cm^−1^ were easily injected without compromising the stability of the Taylor cone. Consequently, many more samples can now qualify for electrospray-based SPI, such as biological macromolecules that are stable only at very low or high ionic strength outside of the conventional electrospray parameter space. In addition, organic and inorganic samples with narrow nonpolar solvent requirements can now be explored, such as for instances select perovskites (Zhang *et al.*, 2022 ▸).

Future work should analyze mixing between both liquids that form the Taylor cone in more detail. Considering the volume of the Taylor cone and the applied flow rates, we estimate the average residence time of liquid in the cone to be approximately 0.1–0.4 ms and therefore comparable withcoaxial- sheet jet formation in double flow focusing nozzles (Oberthuer *et al.*, 2017 ▸). Ammonium acetate or ethanol with a diffusion coefficient in water of order 10^−9^ m^2^ s^−1^ diffuses a distance of approximately 0.5–1 µm during this time span. Accordingly, significant mixing can be expected in sub-micrometre diameter jets. Furthermore, circulation eddies have been reported to occur in the Taylor cone tip (Kim *et al.*, 2020 ▸), possibly resulting in more complex mixing patterns.

Qualification of new samples for CHeES will hence likely require extensive prior testing to identify optimal sample buffer and liquid-sheet conditions. For example, when a biological macromolecule is sensitive to ethanol, an optimal low-ethanol or even ethanol-free sheet-liquid has to be identified first. Such validation work should confirm sample integrity via transmission electron microscopy after spraying (Bielecki *et al.*, 2019 ▸; Yang *et al.*, 2024 ▸). In turn, time-resolved mixing experiments may benefit from increased mixing through dedicated 3D micromixer structures (Knoška *et al.*, 2020 ▸). With these aspects CHeES may also help advance other imaging modalities, such as for instance electrospray-assisted electron microscopy (Yang *et al.*, 2024 ▸).

## Supplementary Material

Coaxial-sheet-liquid optimization for CHeES Taylor cone stability. Among the tested range of 20-120 mM ammonium acetate, a 80 mM ammonium acetate in ethanol showed optimal Taylor cone stability and aerosol generation when injected at 401 nL/min. DOI: 10.1107/S1600577525003686/gy5069sup1.mp4

Supplementary Materials: Coaxial Helium Electrospray for Single-Particle Imaging at X-ray Free Electron Lasers. DOI: 10.1107/S1600577525003686/gy5069sup2.pdf

## Figures and Tables

**Table 1:** Experimental gas-sheet and coaxial-liquid-sheet flow rates in helium and nitrogen mode

Parameter	Helium mode		Nitrogen mode
Coaxial-liquid-sheet	Water-based	Ethanol-based	Ethanol-based
He flow (L min^−1^)	3	3	–
N_2_ flow (L min^−1^)	0.08	0.06	1
CO_2_ flow (L min^−1^)	0.06	0.03	0.13
Applied voltage (V)	3900–4400	1650–1950	1800–2100
Liquid flow (nL min^−1^)	≥357	≥433	≥433
Liquid conductivity (µS cm^−1^)	503	659	659
Ammonium acetate (m*M*)	5	80	80
