# Supplementary material for: Coaxial helium electrospray for single-particle imaging at X-ray free electron lasers
Source: J Synchrotron Radiat. 2025 Jun 6;32(Pt 4):849–60. doi: 10.1107/S1600577525003686 (PMC12236250; doi:10.1107/S1600577525003686)
Supplement: Supplementary file 2 [file s-32-00849-sup2.pdf]

Supplementary Materials: Coaxial Helium Electrospray for Single-Particle Imaging at X-ray Free Electron Lasers

Safi Rafie-Zinedine[1,2], Joachim Schulz[1], Johan Bielecki[1], and Michael Heymann[2]

[1] *European XFEL, Holzkoppel 4, 22869 Schenefeld, Germany*
[2] *Institute of Biomaterials and Biomolecular Systems, University of Stuttgart, Pfaffenwaldring 57, Stuttgart, 70569, Germany*

1 Sample and Buffer Preparation

Ethanol-based with 20 to 120 mM ammonium acetate was diluted from a 500 ml stock solution with 0.5 M ammonium acetate in water stock. Water-based buffers with 2 to 500 mM ammonium acetate were diluted from a 500 ml stock solution with 1 M ammonium acetate in water stock. The dilutions were performed twice, once against water, and in addition by adding sucrose to a final concentration of 1% (v / v). In addition, a 1% sucrose solution was prepared in ultrapure water without ammonium acetate.

2 Configuration of Finite-Element Model

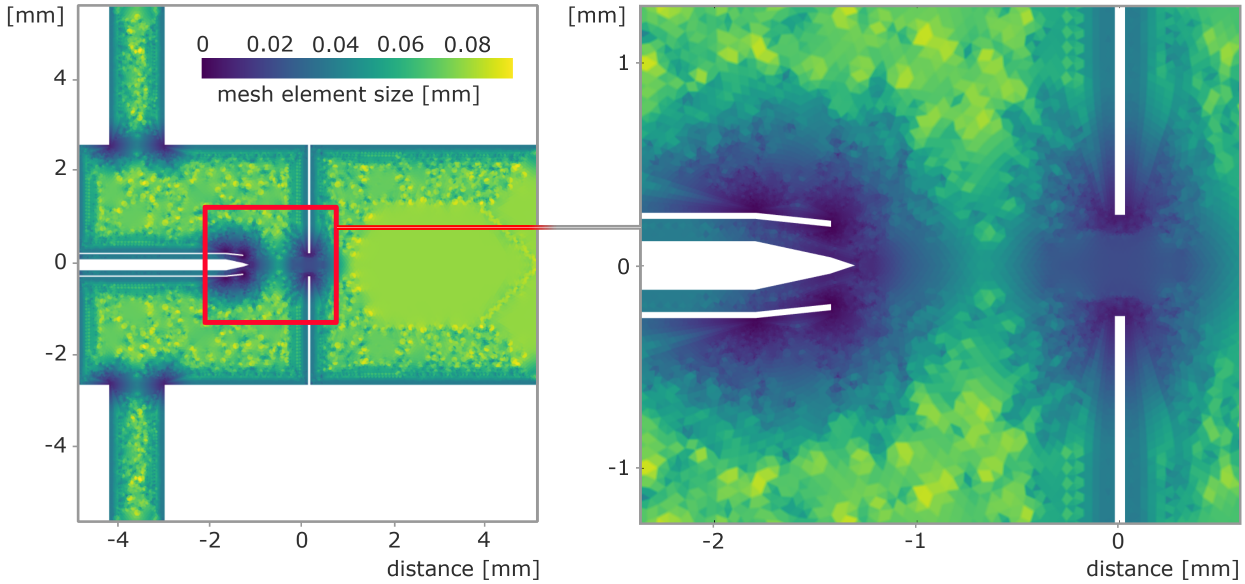

SI-Figure 1: The element size of the mesh in the aerosolization and neutralization chambers within the CHeES system, with a zoom-in on the area around the Taylor cone.

3 CHeES Design

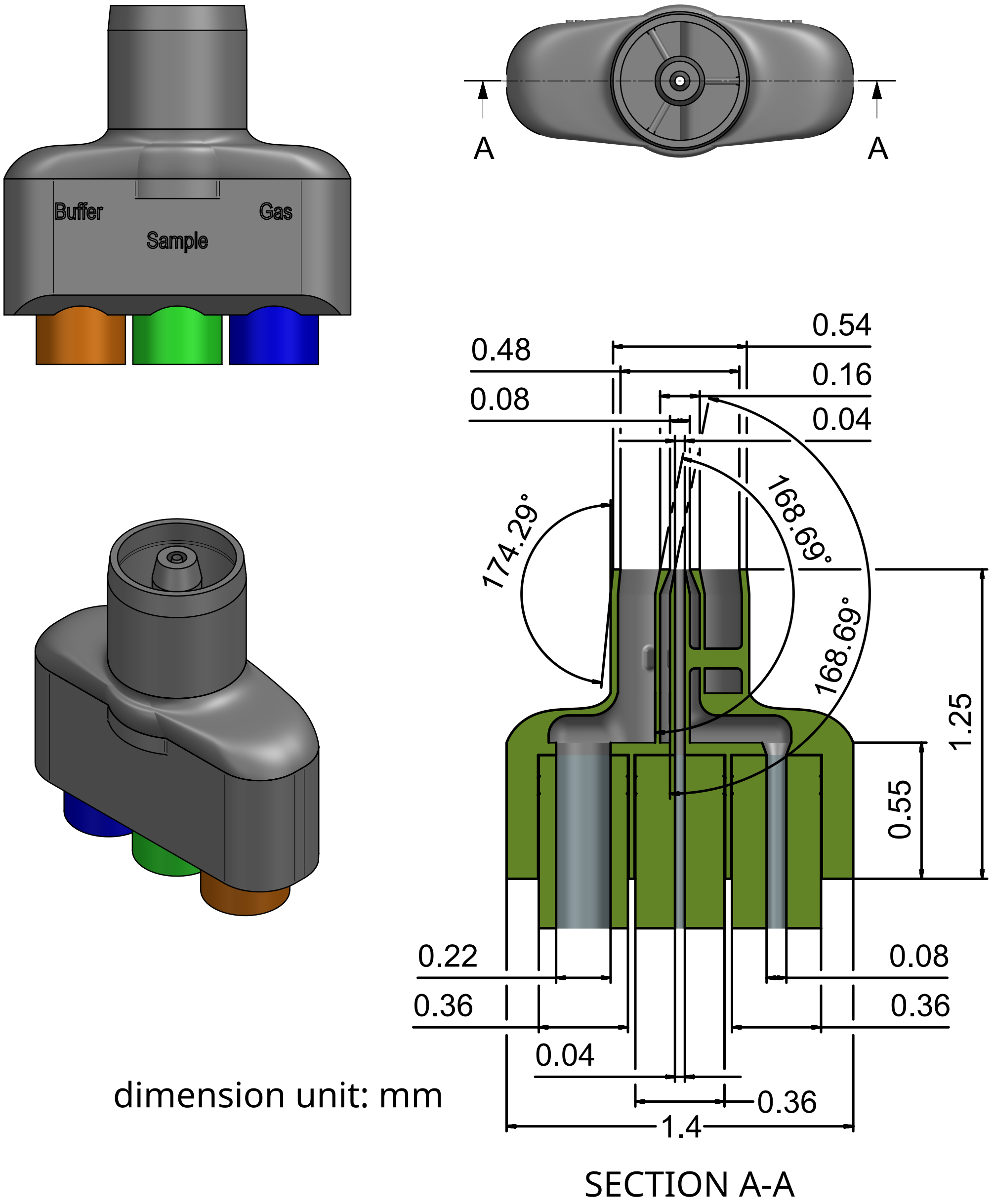

SI-Figure 2: CHeES nozzle design. Renderings and technical drawing to nozzle dimensions, inlets, and outlets. Dimensions in milimeter.

4 Simulations of Gas Flow Around the Taylor Cone

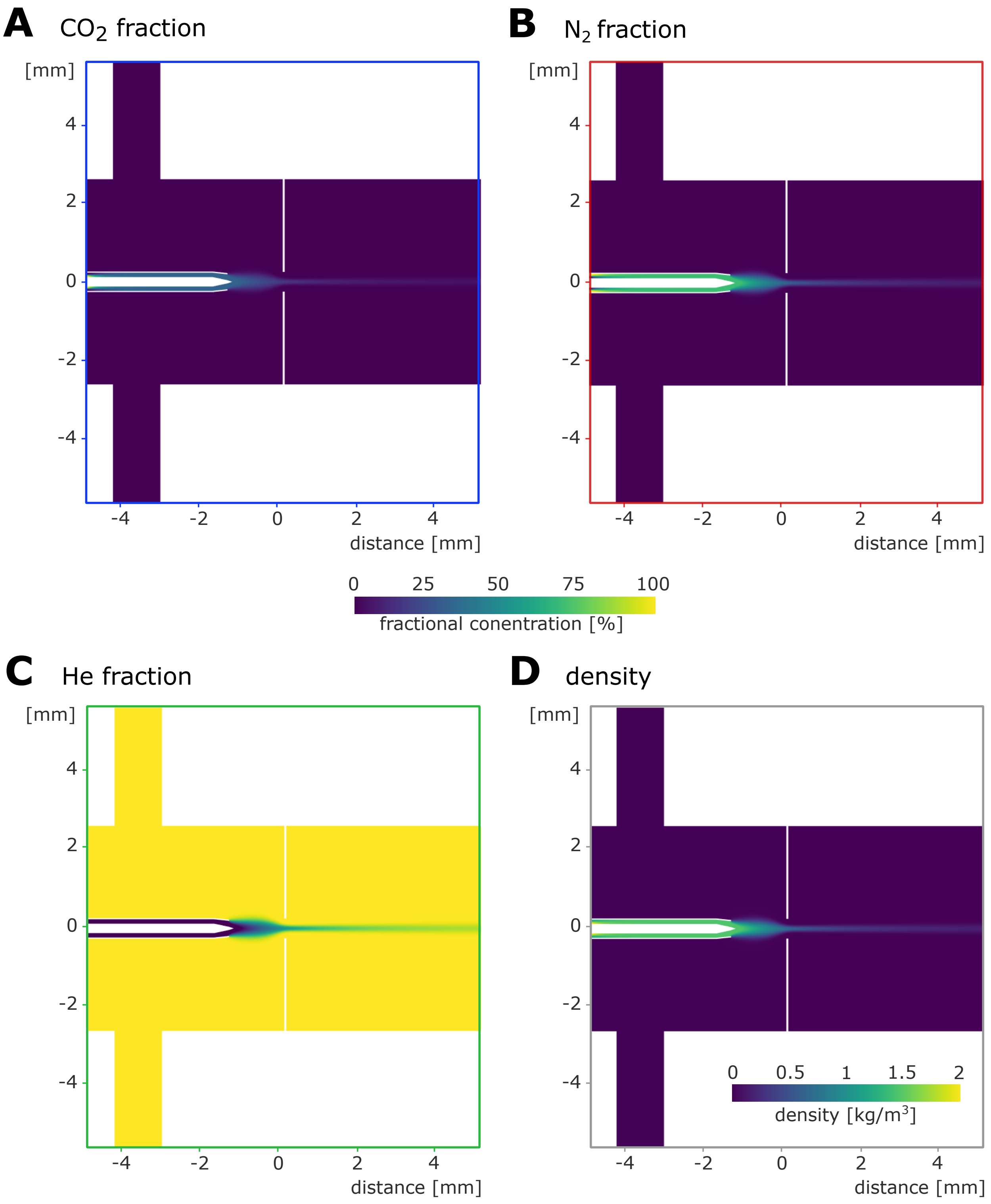

SI-Figure 3: Finite element modeling of fractional gas concentrations of (A) CO2, (B) N2, (C) He, and (D) the gas density in the aerosolization and neutralization chambers within the CHeES system.

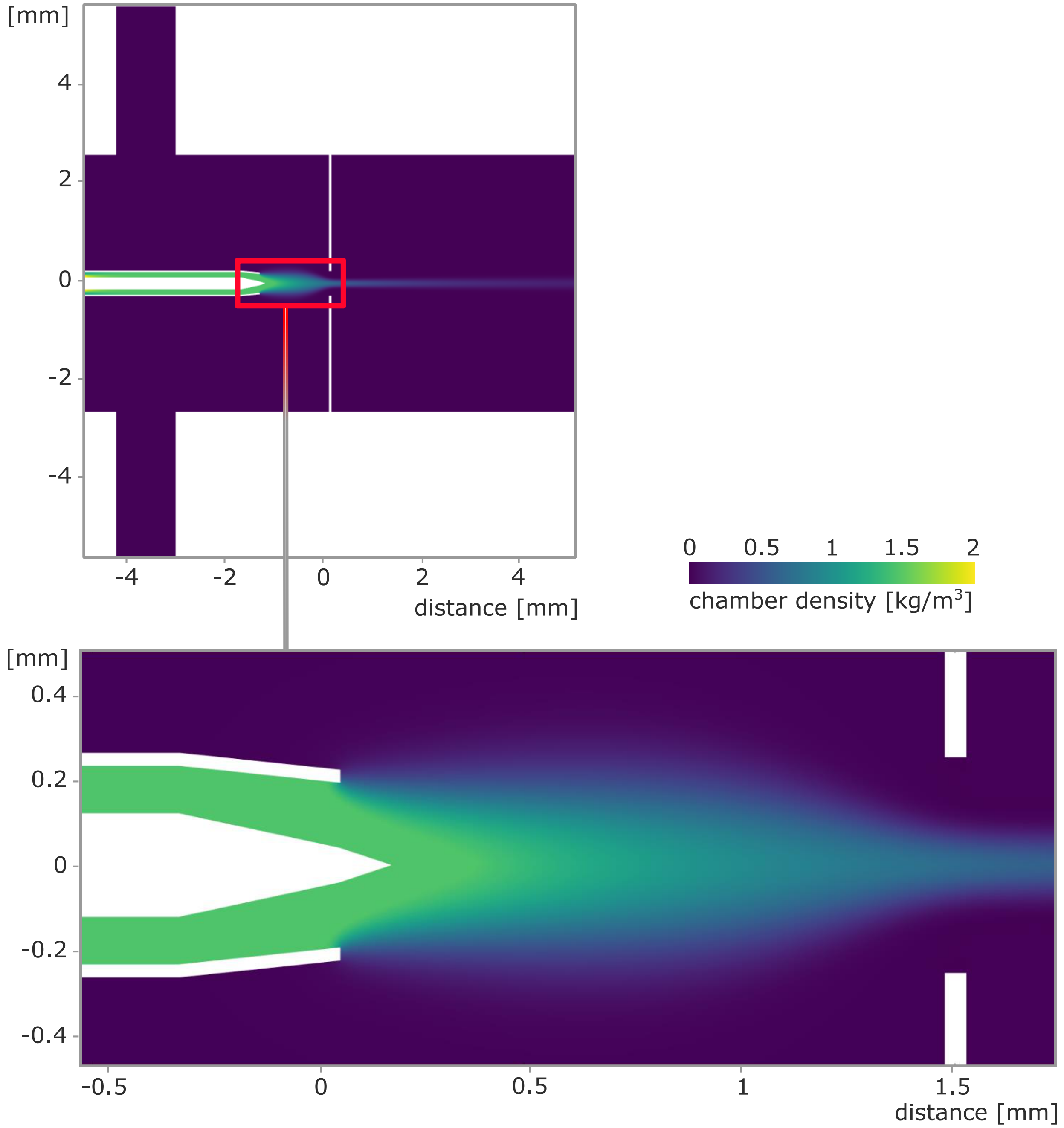

SI-Figure 4: The gas density in the aerosolization and neutralization chambers within the CHeES system, with a zoom-in on the area around the Taylor cone.

5 HeES gas modes

SI-Table 1: He-mode and N_2-mode operation conditions for HeES injection.

Parameter	He-mode	N_2-mode
He flow [L/min]	4.2	–
N_2 flow [L/min]	0.03	1
CO_2 flow [L/min]	0.015	0.2
applied Voltage [V]	2200 - 2600	NA
liquid flow rate [nL/min]	100 - 200	100 - 200

6 Conductivity range quantification

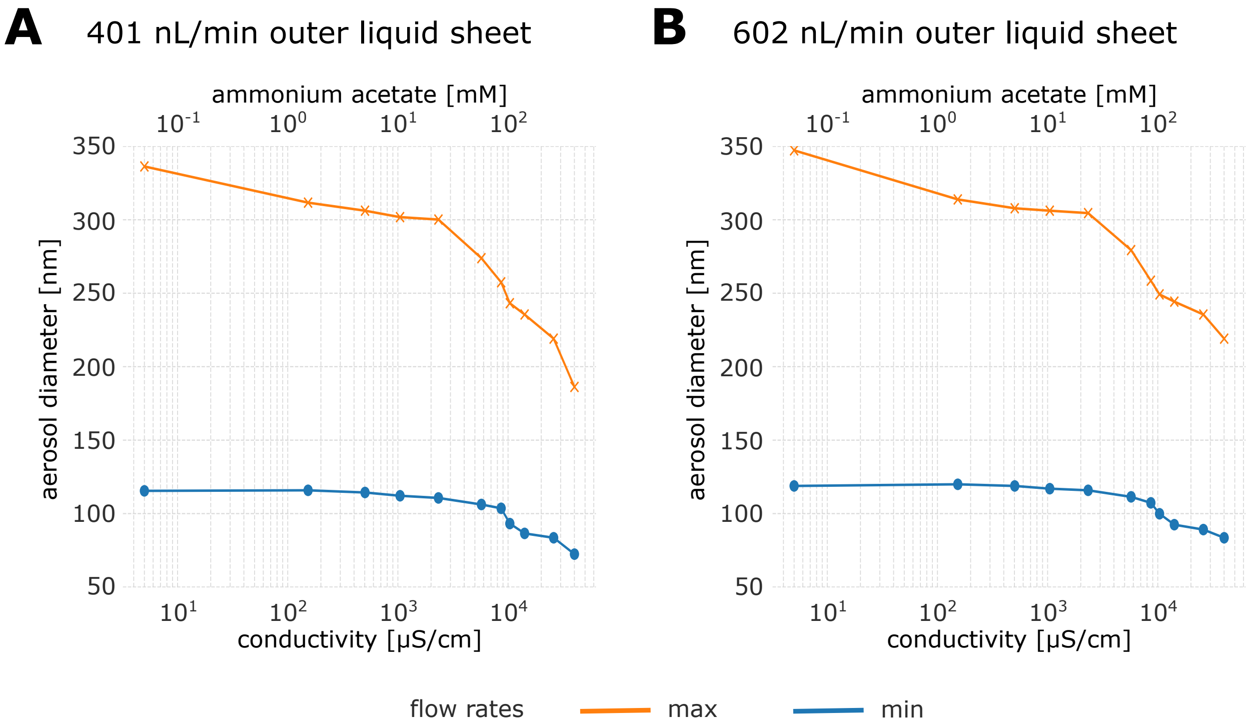

SI-Figure 5: Droplet size as a function of conductivity, showing a decrease in droplet size with increasing conductivity: with an outer liquid flow of (A) 401 nL/min, and (B) 602 nL/min.

7 Core-shell Analysis

SI-Table 2: Inference of droplet structure produced by the CHeES system following the procedure of the core-shell analysis from Figure 5.6. Using the six marked conditions from Figure 5.5, we infer the core diameter, shell thickness, and core + shell diameter of the droplets. The table shows that higher surface charge at the Taylor cone produces smaller droplets and thus smaller particles.

Ammonium Acetate	Inner liquid		Outer liquid		Measured particle	Inferred particle diameter [nm]		
buffer background	Sucrose	Flow rate [nL/min]	Sucrose	Flow rate [nL/min]	diameter [nm]	Core diameter [nm]	Shell thickness [nm]	Entire droplet diameter [nm]
	1 wt%		—		25 ± 0.38	116 ± 1.8	—	—
2 mM	—	32	1 wt%	401	47.6 ± 0.45	—	57 ± 2.1	—
	1 wt%		1 wt%		51.1 ± 1	—	—	237 ± 4.6
	1 wt%		—		23.8 ± 0.35	111 ± 1.6	—	—
20 mM	—	32	1 wt%	401	45.2 ± 0.8	—	54 ± 3.7	—
	1 wt%		1 wt%		48.4 ± 0.93	—	—	214 ± 4.3
	1 wt%		—		15.6 ± 0.22	72 ± 1	—	—
500 mM	—	32	1 wt%	401	30.4 ± 0.5	—	37 ± 2.3	—
	1 wt%		1 wt%		32.1 ± 0.7	—	—	149 ± 3.2
	1 wt%		—		25.8 ± 0.5	120 ± 2.3	—	—
2 mM	—	49	1 wt%	602	70.3 ± 1.5	—	106 ± 7	—
	1 wt%		1 wt%		72.9 ± 1.7	—	—	334 ± 7.9
	1 wt%		—		25 ± 0.5	116 ± 2.3	—	—
20 mM	—	49	1 wt%	602	66.1 ± 1.3	—	98 ± 6	—
	1 wt%		1 wt%		68.6 ± 1.3	—	—	297 ± 6
	1 wt%		—		18 ± 0.45	83 ± 2.1	—	—
500 mM	—	49	1 wt%	602	44.2 ± 1	—	63 ± 4.6	—
	1 wt%		1 wt%		46.5 ± 1.1	—	—	208 ± 5.1

8 Background Noise Reduction

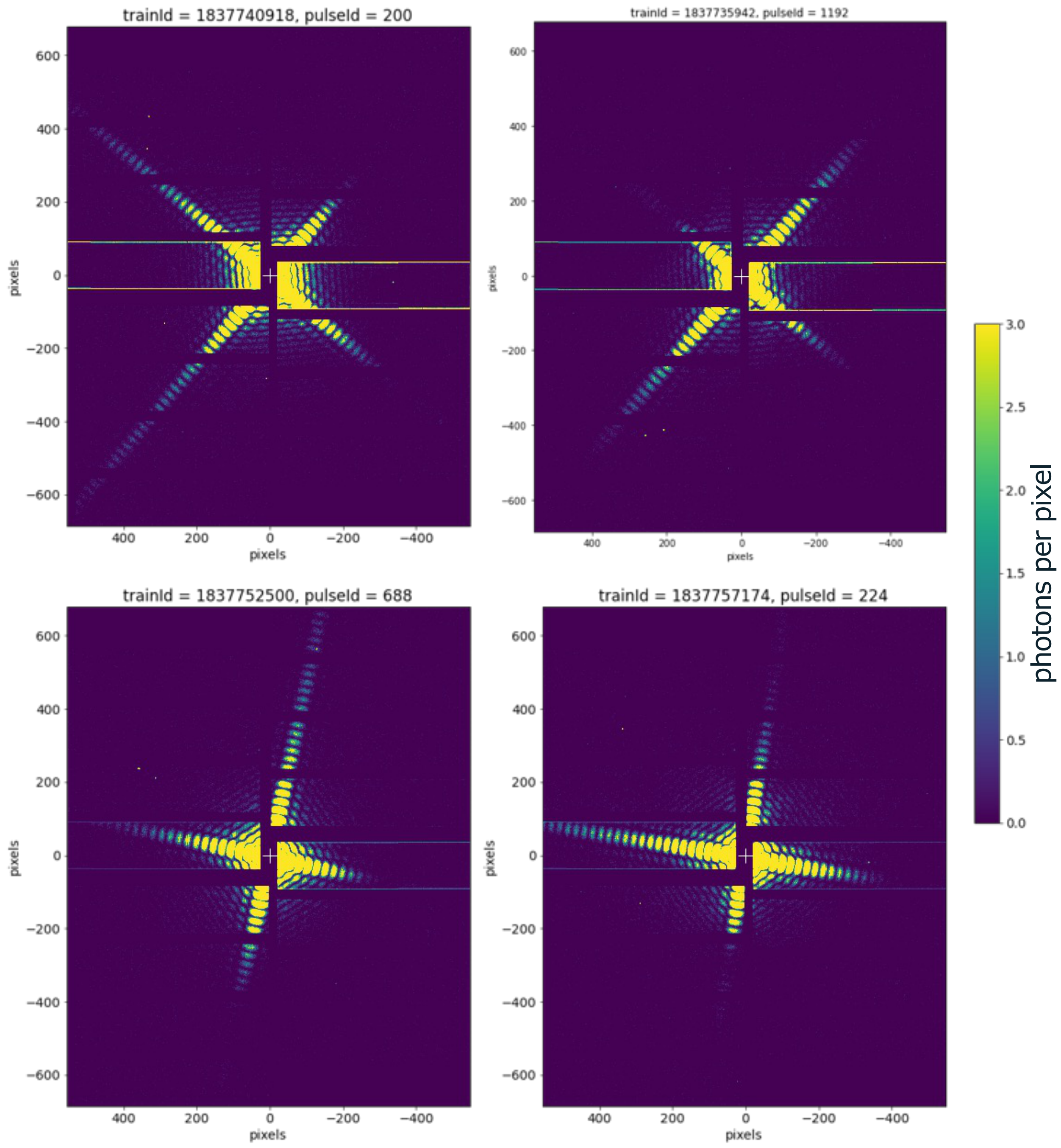

SI-Figure 6: Four high-quality diffraction patterns were collected from cube-shaped silver nanoparticles with a side length of 55 nm at the SPB/SFX instrument at the European XFEL, where silver nanocubes were injected using the CHeES system.
